# Correlation between blood pressure changes and AMS, sleeping quality and exercise upon high-altitude exposure in young Chinese men

**DOI:** 10.1186/2054-9369-1-19

**Published:** 2014-08-26

**Authors:** Yang Liu, Ji-Hang Zhang, Xu-Bin Gao, Xiao-Jing Wu, Jie Yu, Jian-Fei Chen, Shi-Zhu Bian, Xiao-Han Ding, Lan Huang

**Affiliations:** Institute of Cardiovascular Diseases of PLA; Department of Cardiology, Xinqiao Hospital, Third Military Medical University, Chongqing, 400037 China

**Keywords:** Arterial blood pressure changes, High altitude, Acute mountain sickness, Sleep quality, Exercise

## Abstract

**Background:**

Excessive elevation of arterial blood pressure (BP) at high altitude can be detrimental to our health due to acute mountain sickness (AMS) or some AMS symptoms. This prospective and observational study aimed to elucidate blood pressure changes induced by exposure to high-altitude hypoxia and the relationships of these changes with AMS prevalence, AMS severity, sleep quality and exercise condition in healthy young men.

**Methods:**

A prospective observational study was performed in 931 male young adults exposed to high altitude at 3,700 m (Lhasa) from low altitude (LA, 500 m). Blood pressure measurement and AMS symptom questionnaires were performed at LA and on day 1, 3, 5, and 7 of exposure to high altitude. Lake Louise criteria were used to diagnose AMS. Likewise, the Athens Insomnia Scale (AIS) and the Epworth Sleepiness Scale (ESS) were filled out at LA and on day 1, 3, and 7 of exposure to high altitude.

**Results:**

After acute exposure to 3,700 m, diastolic blood pressure (DBP) and mean arterial blood pressure (MABP) rose gradually and continually (*P* < 0.05). Analysis showed a relationship with AMS for only MABP (*P* < 0.05) but not for SBP and DBP (*P* > 0.05). Poor sleeping quality was generally associated with higher SBP or DBP at high altitude, although inconsistent results were obtained at different time (*P* < 0.05). SBP and Pulse BP increased noticeably after high-altitude exercise (*P* < 0.05).

**Conclusions:**

Our data demonstrate notable blood pressure changes under exposure to different high-altitude conditions: 1) BP increased over time. 2) Higher BP generally accompanied poor sleeping quality and higher incidence of AMS. 3) SBP and Pulse BP were higher after high-altitude exercise. Therefore, we should put more effort into monitoring BP after exposure to high altitude in order to guard against excessive increases in BP.

## Background

Two cardinal challenges to life at high altitude are the low ambient temperature and hypobaric hypoxia. Temperature declines approximately 1°C for each 150 m elevation. Barometric pressure also decreases progressively with increasing altitude. And harmful effects of hypoxia are experienced by most maladaptive subjects at high altitude [1, 2]. In response to a short-term hypoxic exposure, blood pressure either does not change or increases modestly, and currently, the consequences are not fully understood. Moreover, most researchers believe that blood pressure (BP) changes at high altitude are principally due to increases in autonomic and sympathetic activity [3–8]. Prolonged hypoxia for up to several days increases systemic pressure gradually, especially diastolic BP (DBP) and mean arterial BP (MABP), in parallel with increases in plasma concentrations of norepinephrine [9, 10]. In particular, excessive elevation of arterial BP is detrimental to our health and can cause acute mountain sickness (AMS) or some AMS symptoms, e.g., headache, dizziness, and insomnia. Some cases may even progress to life-threatening cerebral or pulmonary edema, known as high-altitude cerebral edema (HACE) and high-altitude pulmonary edema (HAPE).

Previous studies on systolic blood pressure (SBP) and diastolic blood pressure (DBP) changes at different altitudes or different time courses of high altitude have been reported, the result is still controversial. One of the purposes of the present study was to confirm the SBP and DBP changes at different altitudes and different time courses of high-altitude exposure and to analyze the MABP and Pulse BP changes. Furthermore, exposure of healthy subjects to high altitude affects arterial BP based on individual factors, the absolute altitude of exposure, the duration of stay at altitude, sleeping quality, and so on. Therefore, the second aim of our study was to investigate blood pressure changes and their relationship with AMS prevalence, AMS severity, sleeping quality and exercise conditions in healthy young men upon high-altitude exposure in order to avoid the risk of hypertension in a high-altitude clinical setting.

## Methods

### Population

Eligible participants had to be non-Tibetan, healthy, young and male lowland residents (18–45 years old). Before entering the high-altitude area, the inclusion criteria were as follows: 1) no organic disease; 2) age ≥18 years; 3) low-altitude dwellers from areas <400 m; and 4) had not been exposed to high altitudes in the previous year. The exclusion criteria were as follows: 1) the presence of autoimmune diseases, respiratory diseases, cardiovascular diseases, malignant tumors, liver and kidney dysfunction, and psychiatric disorders or neuroses that prevented the completion of the questionnaires; 2) age ≥45 years; 3) were from elevations >500 m; 4) were exposed to high altitude in the previous 3 months; or 5) were reluctant to cooperate with the investigation.

### Ethics statement

All participants who agreed to participate in the study were familiar with the purpose and process of this study. The research was approved by the Ethics Committee of Xinqiao Hospital, the Second Clinic Medical College of Third Military Medical University. Before the trial, each participant provided written informed consent and was conscious of his right to withdraw without prejudice at any time. The subjects did not take medication or receive any intervention, and all the data were anonymized prior to retrieval and analysis.

### Randomization

The subjects were randomly assigned to three groups: a low-altitude group (LA), a high-altitude non-exercise group (Day 1, Day 3, Day 5, Day 7) and a high-altitude exercise group (Day 7), using a computer generated random number list.

### Procedure

**Figure 1:**
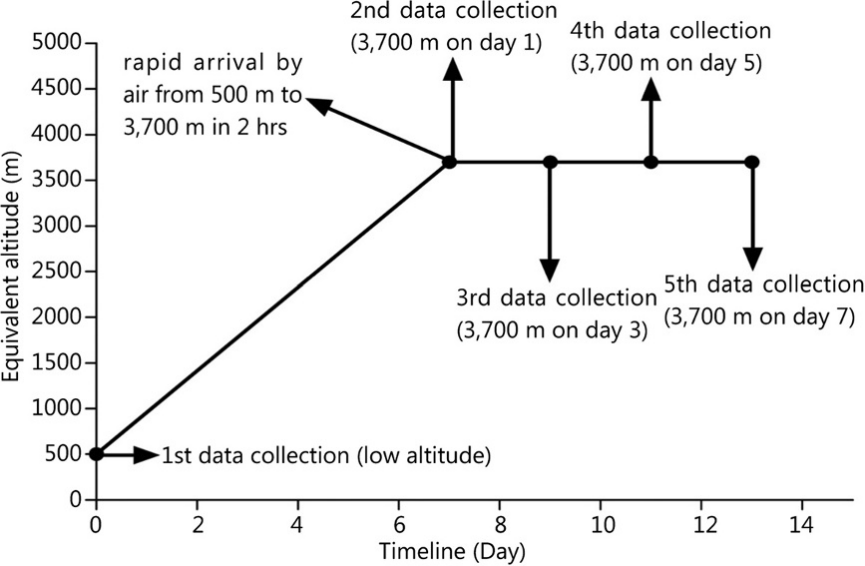
**Data collection schedule.** This research utilized an all-around design on selected young Chinese men for the test condition. The test conditions were defined as LA (500 m, Control group) for baseline testing and abrupt exposure to 3,700 m after approximately a week at LA.

This study was performed at two locations that were separately defined as low altitude (Chongzhou, LA, 500 m) and high altitude (Lhasa, 3,700 m). Low-altitude subjects ascended to 3,700 m after approximately a week on the Chongzhou plain. Participants completed the Lake Louise Score (LLS) AMS self-assessment test, the Athens Insomnia Scale (AIS) questionnaire and the Epworth Sleepiness Scale (ESS) questionnaire. Arterial blood pressure measurements were carried out. All of the trial procedures were performed at 500 m within one week before ascending in Chongzhou and within 24 h after arrival at 3,700 m (in Lhasa, approximately 13:00 pm from June 21st to 25th, 2012, and examinations were performed at approximately 8:00–11:00 am on the next morning upon arrival. The minimal and maximal times from arrival to the examination were 19 hours and 22 hours, respectively). The subjects stayed at 3,700 m for a week before leaving for the next work place. An outline of the study design and testing schedule is shown in Figure 1.

### Outcome measures

The primary outcome measure was the change of blood pressure. The secondary outcome measures were as follows: the incidence of acute mountain sickness at altitude; its severity reflected by the LLS score; SaO_2_; sleep quality assessed by questionnaires; and age (y/years), weight (W/g), and height (H/cm) according to the AMS symptoms Questionnaire. The body mass index (BMI) was calculated as weight in kilograms divided by the square of height in meters.

#### Blood pressure measurement

All selected subjects, both at low altitude and high altitude, were on the same standard diet, which included a fixed amount of daily proteins, carbohydrates, and fat, but the measurements were performed after a 4-hour fast and at least an 8-hour abstinence from caffeine and a 24-hour abstinence from alcohol. After the subjects had been seated in a chair at rest for at least 15 minutes, non-invasive measurements of resting systolic blood pressure (SBP) and diastolic blood pressure (DBP) were obtained by a wrist sphygmomanometer (OMRON HEM-6,200) at the same time of day. All subjects completed the full study at altitude. Optimal blood pressure was defined as a SBP <120 mmHg and a DBP <80 mmHg. Prehypertension was defined at a SBP of 120 to 139 mmHg and/or a DBP of 80 to 89 mmHg, and hypertension was diagnosed at a SBP ≥140 mmHg and/or a DBP ≥90 mmHg, according to international guidelines [11, 12]. MABP (mean arterial blood pressure) values were calculated from SBP and DBP values with the following equation: MABP = [(SBP-DBP)/3] + DBP; Pulse BP (Pulse blood pressure) values were also calculated from SBP and DBP values with the following equation: Pulse BP = SBP-DBP.

#### Oxygen saturation (SO_2_) measurement

The second day after the subjects arrived at the destination, SaO2 was measured by Pulse Oximeter (NONIN-9550, Nonin Onyx, America) in triplicate after the subjects had rested in a seated position for 15 minutes.

#### Acute mountain sickness assessment

Acute mountain sickness was diagnosed by the Lake Louise Scoring System (LLS). This is a five-item self-administered questionnaire on the basis of the most frequent symptoms of AMS: headache, gastrointestinal problems (anorexia, nausea, or vomiting), insomnia, weakness or fatigue, and dizziness or light-headedness. Every item is scored by the subject on a scale from 0 to 3, with each integer having a specific descriptor. The minimum LLS score is 0, and the maximum score is 15; clinical AMS was diagnosed when headache and one or more other symptoms occurred and reached a Lake Louise score of ≥3 (range, 0 to 15) at any time point. Severity was assessed according to the following categories: mild (3–4), moderate (5–10), and severe (11–15) [13].

#### Epworth Sleepiness Scale (ESS) assessment

The Epworth Sleepiness Scale [14, 15] is a measurement of daytime somnolence that includes eight items. Items 1 to 8 are as follows: (1) sitting and reading, (2) watching television, (3) sitting inactive in a public place (e.g., a theater or meeting), (4) sitting as a passenger in a car for an hour without a break, (5) lying down to rest in the afternoon when circumstances permit, (6) sitting and talking to someone, (7) sitting quietly after a lunch without alcohol, (8) sitting in a car while stopped for a few minutes in traffic. Each item is scored as 0 to 3, where 0 represents would never doze, 1 represents a slight chance of dozing, 2 represents a moderate chance of dozing, and 3 represents a high chance of dozing. The ESS score is the sum of items 1 to 8.

#### Athens Insomnia Scale (AIS) assessment

The AIS [15, 16] also includes eight items: 1) difficulty in sleep duration, 2) awakening during the night, 3) final awakening earlier than desired, 4) insufficient total sleep duration, 5) dissatisfaction with overall quality of sleep, 6) decreased sense of wellbeing during the day, 7) decreased functioning during the day, and 8) sleepiness during the day. Each item is measured on a 4-point Likert scale. A total score of 6 or higher is recognized as insomnia [15, 17].

#### Exercise condition

At high altitude, before the first step test, blood pressure was recorded (Pre-exercise). According to the velocity control of the metronome at 30 times per minute, subjects began to do the first step test with 0.3-meter-high steps for 5 minutes, and then, the first measure of blood pressure was recorded immediately after exercise [Post-exercise (1)]. After a rest for 5 minutes, subjects continued to do the second step test at 0.3-meter-high steps for 5 minutes, and then, the second measure of blood pressure was recorded immediately after exercise [Post-exercise (2)].

### Statistical analysis

All analyses were conducted using the SPSS 19.0 software (Chicago, IL, USA). The results were presented as the mean ± standard deviation. To apply parametric tests dealing with continuous variables, we assessed the normality of distributions using the one-sample Kolmogorov-Smirnov test. Data were analyzed using nonparametric statistics for non-normally distributed variables. Four variables of time (SBP, DBP, MABP and Pulse BP) were estimated for each station as well as the between-subject changes using a repeated measure ANOVA and the Kruskal-Wallis *H* test. Differences in the mean values between the two groups of subjects with and without AMS were compared by the independent-samples *t*-test or the Mann–Whitney test. The significance level was established at *P*-value <0.05.

## Results

We collected 931 AMS symptom questionnaires (excluding 40 lost follow-up and 23 uncompleted) valid at both 500 m and 3,700 m. General basal features in the studied group were rather homogeneous (*P* > 0.05).

### The incidence of acute mountain sickness

On day 1, 3, 5, and 7 at 3,700 m, AMS was present in 62.75% (128/204), 20.97% (13/62), 25% (14/56), and 13.33% (8/60) of subjects.

### Blood pressure

#### Systolic blood pressure

**Figure 2:**
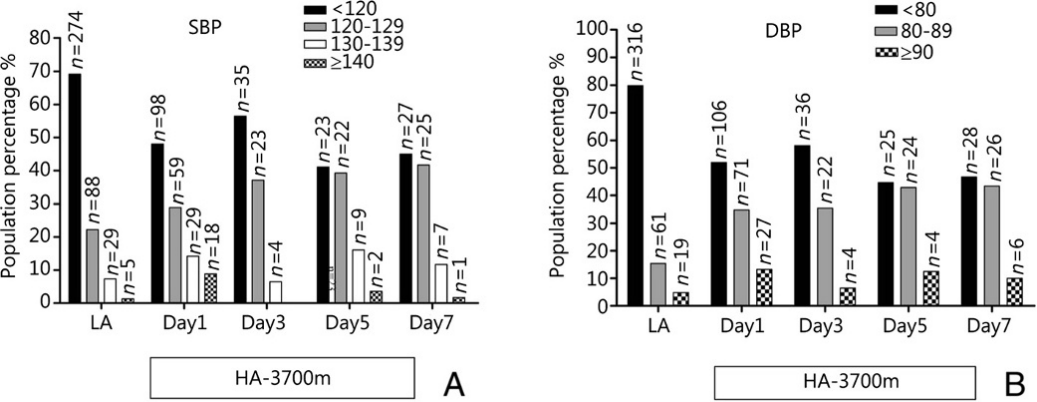
**Interclass distribution of arterial blood pressure at different altitudes and time courses. (A)** SBP (systolic blood pressure, mmHg), **(B)** DBP (diastolic blood pressure, mmHg). LA: Low altitude (500 m); HA-3,700 m: High-altitude 3,700 m. Day 1, Day 3, Day 5, Day 7: all at high-altitude 3,700 m. Values are proportions. ^a^ *P* < 0.05 compared with LA.

1. Altitudes: On day 1 at 3,700 m, high-altitude SBP (121.25 ± 12.69 mmHg) was significantly higher than that at LA (115.15 ± 10.53 mmHg, *P* = 0.000, Table 1). Although the mean SBP value at 3,700 m (Day 1) was within normal range, an interclass analysis illustrated that a proportion of the subjects (51.96%) with SBP values over 120 mmHg was higher than that at LA (30.81%, Figure 2). Moreover, the mean SBP value of over 120 mmHg at 3,700 m (Day 1) (129.98 ± 10.07 mmHg) was higher than that at LA (127.42 ± 7.99 mmHg, *P* = 0.037).
2. Time courses: After acute exposure to 3,700 m (Day 1), SBP rose noticeably and then slightly decreased on Day 3 (*P* > 0.05). Thereafter, it began to slightly increase again on Day 5 (*P* > 0.05, Table 1). Although the mean SBP values on day 1, 3, 5, and 7 at 3,700 m were within normal ranges, an interclass analysis indicated a high proportion of the subjects on day 5 and 7 at 3,700 m (>50%) with SBP values of over 120 mmHg (Figure 2). The mean SBP value of over 120 mmHg on day 1 at 3,700 m (129.98 ± 10.07 mmHg) was higher than that on day 3 at 3,700 m (125.19 ± 4.85 mmHg, *P* = 0.001) while it was similar to that on day 5 and 7 at 3,700 m (127.21 ± 5.90 and 126.42 ± 6.47 mmHg; *P* = 0.054, *P* = 0.059, respectively).
3. AMS prevalence: There was no difference in the SBP between the AMS and non-AMS groups at any period (*P* > 0.05, Table 2).
4. AMS severity: On day 1 at 3,700 m, SBP was higher in the severe AMS group than that in the mild or moderate AMS group (*P* = 0.033, *P* = 0.013, respectively, Table 3).
5. Sleep quality: On day 7 at 3,700 m, SBP in the sleepiness group was higher than that in the non-sleepiness group (*P* = 0.007).
6. Exercise group: Compared with the Low altitude SBP (EG1), the Post-exercise (2) SBP increased at 3,700 m (Day 2, *P* = 0.001, Table 4). Compared with the Pre-exercise SBP, the Post-exercise (1) and Post-exercise (2) SBPs were higher (*P* = 0.006, *P* = 0.000). Compared with the Low altitude SBP (EG2), the Pre-exercise, Post-exercise (1) and Post-exercise (2) SBPs were higher (*P* = 0.045, *P* = 0.001, *P* = 0.000) at 3,700 m (Day 7), and the Post-exercise (2) SBP was higher than the Pre-exercise SBP (*P* = 0.009).

**Table 1:** **Time course of blood pressure changes (mmHg,** ***x*** **±** ***s*** **)**

Index	LA	High altitude 3,700 m			
		Day 1	Day 3	Day 5	Day 7
SBP	115.15 ± 10.53	121.25 ± 12.69^a^	116.47 ± 10.07	121.02 ± 9.58	119.75 ± 9.36
DBP	72.50 ± 9.56	79.41 ± 9.45^a^	79.18 ± 9.96^a^	81.04 ± 8.80^a^	78.48 ± 9.11^a^
MABP	86.71 ± 8.93	93.36 ± 9.55^a^	90.47 ± 8.47^a^	94.36 ± 8.56^a^	92.24 ± 7.81^a^
Pulse BP	42.65 ± 8.06	41.84 ± 9.98^a^	39.00 ± 8.04^a^	39.98 ± 6.43^a^	41.27 ± 10.28

SBP, Systolic blood pressure; DBP, Diastolic blood pressure; MABP, Mean arterial blood pressure; Pulse BP, Pulse blood pressure. Values are means ± SD (standard deviation). ^a^ *P* < 0.05 compared with LA.

**Table 2:** **Blood pressure, SaO**_**2**_ **and AMS at HA-3700 m Day 1 (mmHg,** ***x*** **±** ***s*** **,** ***n*** **= 204)**

Grade	SBP	DBP	MABP	Pulse BP	SaO _2_
Non-AMS	118.77 ± 11.00	77.76 ± 10.08	91.43 ± 9.56	41.01 ± 8.67	88.88 ± 3.77
AMS	122.69 ± 13.41	80.37 ± 8.97	94.48 ± 9.40	42.32 ± 10.67	87.66 ± 3.64
*P* value	0.068	0.057	0.028	0.699	0.024

HA-3,700 m: High-altitude 3,700 m. Values are means ± SD (standard deviation).

**Table 3:** **Blood pressure, SaO**_**2**_ **and severity of AMS at HA-3,700 m Day 1 (mmHg,%,** ***x*** **±** ***s*** **,** ***n*** **= 204)**

Grade	SBP (mmHg)	DBP (mmHg)	MABP (mmHg)	Pulse BP (mmHg)	SaO _2_(%)
Mild AMS	122.89 ± 13.98	79.44 ± 8.89	93.92 ± 9.18	43.45 ± 12.27	87.37 ± 3.67
Moderate AMS	121.73 ± 12.43	81.14 ± 8.97	94.67 ± 9.51	40.59 ± 8.14	87.93 ± 3.57
Severe AMS	144.50 ± 4.94^ab^	89.50 ± 2.12	107.83 ± 0.24^a^	55.00 ± 7.07^b^	90.5 ± 4.95

HA-3,700 m: High-altitude 3,700 m. Values are means ± SD (standard deviation). ^a^ *P* < 0.05 compared with mild AMS. ^b^ *P* < 0.05 compared with moderate AMS.

**Table 4:** **High-altitude blood pressure changes and exercise (mmHg,** ***x*** **±** ***s*** **)**

Item			SBP (mmHg)	DBP (mmHg)	MABP (mmHg)	Pulse BP (mmHg)
EG1	3,700 m Day 2 (*n* = 100)	Low altitude (*n* = 100)	117.59 ± 11.75	76.44 ± 11.08	90.19 ± 10.80	41.10 ± 6.92
		Pre-exercise	114.76 ± 11.15	75.44 ± 8.98	88.55 ± 8.85	39.32 ± 8.71
		Post-exercise (1)	119.61 ± 13.32^b^	77.37 ± 10.28	91.45 ± 10.06^b^	42.24 ± 11.28^b^
		Post-exercise (2)	123.13 ± 12.47^ab^	75.46 ± 11.15	91.35 ± 10.08^b^	47.67 ± 12.21^ab^
EG2	3,700 m Day 7 (*n* = 53)	Low altitude (*n* = 53)	111.60 ± 10.15	70.00 ± 8.09	83.87 ± 8.16	41.60 ± 7.14
		Pre-exercise	116.08 ± 12.45^c^	74.38 ± 10.96^c^	88.28 ± 10.26^c^	41.70 ± 10.90
		Post-exercise (1)	120.26 ± 15.77^c^	75.89 ± 11.71^c^	90.68 ± 12.26^c^	44.38 ± 10.41
		Post-exercise (2)	122.38 ± 11.97^cd^	75.26 ± 11.76^c^	90.97 ± 10.37^c^	47.11 ± 12.08^cd^

EG: Exercise Group. Pre-exercise: before the first exercise at high-altitude 3,700 m; Post-exercise (1): after the first exercise at high-altitude 3,700 m; Post-exercise (2): after the second exercise. ^a^ *P* < 0.05 compared with low altitude (EG1). ^b^ *P* < 0.05 compared with high-altitude 3,700 m Day 2 (EG1) Pre-exercise. ^c^ *P* < 0.05 compared with Low altitude (EG2). ^d^ *P* < 0.05 compared with high-altitude 3,700 m Day 7 (EG2) Pre-exercise.

#### Diastolic blood pressure

1. Altitudes: On day 1 at 3,700 m, DBP (79.41 ± 9.45 mmHg) was higher than that at LA (72.50 ± 9.50 mmHg) (*P* = 0.000, Table 1). Although the mean DBP value on day 1 at 3,700 m was within normal range, an interclass analysis showed that the proportion of the subjects with DBP values over 120 mmHg on day 1 at 3,700 m (48.04%) was higher than that at LA (30.81%, Figure 2).
2. Time courses: After acute exposure to 3,700 m (Day 1), DBP escalated noticeably and, thereafter, increased gradually and persistently, above the level of LA (*P* > 0.05, Table 1). Although the mean DBP values on day 1, 3, 5, and 7 at 3,700 m were within normal ranges, an interclass analysis indicated that a high proportion of the subjects had DBP values of over 80 mmHg on day 5 (55.36%) and day 7 (53.33%) at 3,700 m (Figure 2). The mean DBP value of over 80 mmHg on day 1 at 3,700 m (87.06 ± 5.67 mmHg) was higher than that on day 3 (84.65 ± 4.88 mmHg) (*P* = 0.049) while it was similar to that on day 5 and 7 (86.77 ± 5.47 and 85.03 ± 4.55 mmHg) (*P* = 0.805; *P* = 0.068, respectively).
3. AMS: At no period did we find an association of DBP values with AMS (*P* > 0.05, Table 2).
4. AMS severity: On day 1 at 3,700 m, DBP showed no differences among the three AMS subgroups (*P* > 0.05, respectively, Table 3).
5. Sleep quality: On day 1 and 3 at 3,700 m, DBP in the insomnia group was higher than that in the non-insomnia group (*P* = 0.049, *P* = 0.024, respectively).
6. Exercise group: At 3,700 m (Day 2, Day 7), there was no difference in DBP between the low altitude, Pre-exercise, Post-exercise (1) and Post-exercise (2) groups (*P* > 0.05, Table 4).

#### Mean arterial blood pressure

1. Altitudes: On day 1 at 3,700 m, MABP (93.36 ± 9.55 mmHg) was noticeably higher than that at LA (86.71 ± 8.93 mmHg, *P* = 0.000, Table 1).
2. Time courses: After acute exposure to 3,700 m (Day 1), MABP increased rapidly, and remained persistently elevated. Its change was analogous to DBP (Table 1).
3. AMS: MABP on day 1 at 3,700 m was much higher in the AMS group (*P* = 0.028). See Table 2.
4. AMS severity: On day 1 at 3,700 m, MABP was higher in the severe AMS group than that in the mild AMS group (*P* = 0.000). See Table 3.
5. Sleep quality: High-altitude MABP showed no difference between the insomnia and non-insomnia groups and between the sleepiness and non-sleepiness groups (*P* > 0.05).
6. Exercise group: On day 2 at 3,700 m, the Post-exercise (1) and Post-exercise (2) MABPs were higher than the Pre-exercise MABP (*P* = 0.031, *P* = 0.038, Table 4). On day 7, the Pre-exercise, Post-exercise (1) and Post-exercise (2) MABPs were higher than the low altitude (EG2) MABP (*P* = 0.016, *P* = 0.001, *P* = 0.000).

#### Pulse blood pressure

1. Altitudes: After acute exposure to 3,700 m, the Pulse BP (41.84 ± 9.98 mmHg) was below that at LA (42.65 ± 8.06 mmHg, *P* = 0.000, Table 1).
2. Time courses: On day 1 at 3,700 m, the Pulse BP was lower than that at LA, and for approximately a week, it remained below that at LA (Table 1).
3. AMS: At no period did we find an association of the Pulse BP between the AMS and non-AMS groups (*P* > 0.05, Table 2).
4. AMS severity: On day 1 at 3,700 m, the Pulse BP was lower in the severe AMS group than that in the moderate AMS group (*P* = 0.017, Table 3).
5. Sleep quality: The High-altitude Pulse BP showed no difference between the insomnia and non-insomnia groups and between the sleepiness and non-sleepiness groups (*P* > 0.05).
6. Exercise group: Compared with low altitude (EG1), the Post-exercise (2) Pulse BP at high altitude was higher (*P* = 0.000); the Post-exercise (1) and Post-exercise (2) Pulse BPs were higher than the Pre-exercise Pulse BP (*P* = 0.042, *P* = 0.000). On Day 7, the Post-exercise (2) Pulse BP was higher than the Pre-exercise BP (*P* = 0.017, Table 4).

### SaO_2_

The SaO_2_ level on day 1 was much higher in the AMS group than that in the non-AMS group (*P* = 0.024). There was no difference in the SaO_2_ level between the mild, moderate and severe AMS groups (*P* > 0.05). See Tables 2 and 3.

## Discussion

This study principally contributes to the comprehensive knowledge of BP changes in a relatively large number of young male subjects during high-altitude exposure. The initial phase of exposure to altitude was connected with a noticeable rise in systolic and diastolic blood pressure and has been noted in other studies [8, 18].

SBP increases as an acute phenomenon on account of an increased and dominant sympathetic activation by hypoxic stress, which is congruous with some reports [7, 19, 20]. It was reported that an elevation of SBP tends to normalize or decrease after a few days at altitude [21–24], which was observed in the current study. Furthermore, our research did not show that any SBP value had an association with AMS; however, in the severe AMS group, SBP was higher.

The DBP analysis is highly relevant for young adults, and this seems to be the most important BP component [21]. Our results show that DBP behaves variably on different occasions. Other researchers have investigated the relationship of BP behavior with altitude, age, and gender in acute hypoxic environments. According to the results of our study, DBP at rest increased gradually with time after high-altitude exposure. This phenomenon has also been reported by other studies [2, 10, 25, 26]. The explanations for the sustained DBP increase observed after more than a week at 3,700 m may chiefly be a consequence of a persistent sympathetic stimulation [21, 27]. This indicates that hypoxia may be a continual stimulus for an organism, as suggested by Siques *et al*. [21], who demonstrated a relationship between lower SaO_2_ values and hypertensive DBP values. Furthermore, the increase of sympathetic tone may be a natural response by non-adapted subjects to counteract the effects of hypoxia. Indeed, hypoxia directly affects the vascular tone of systemic resistance vessels and increases ventilation and sympathetic activity via the stimulation of peripheral chemoreceptors [24, 26]. Interactions occur between the hypoxic vasodilatation of systemic arterioles and the chemoreceptor-mediated responses in the systemic circulation [2, 21, 28]. This, in turn, leads to baroreceptor-mediated sympathetic excitation. Alterations in baroreflex function, an increase in the “set point” and possibly a decrease in gain, are also likely to contribute. These autonomic adaptations may have a role in an escalation in BP during sustained hypoxia [2, 29]. It has been reported that hypoxia-induced hypertension is linked with a transient rise in plasma endothelin and a depressed production of nitric oxide in rats [2, 30]. Moreover, the subsequent decrease of DBP is potentially secondary to the circulation of hypoxia-induced inflammatory markers that have vasodilating properties and cause an overall reduction in DBP.

The change pattern of MABP bears a resemblance to that of DBP. Acute exposure to altitude was associated with a rise in MABP. Sizlan *et al*. [2] highlighted the gradual increase in MABP at rest with time at altitude, which was also observed in other studies [2, 10, 19, 31]. This also occurred in our subjects. Our research demonstrated that blood pressure tends to be higher in the AMS group, especially MABP, as was previously reported by Beidleman *et al*. [32]. The mechanism of this relationship between blood pressure and AMS could be associated with an exaggeration in sympathetic tone that causes peripheral vasoconstriction and, thus, an increase in blood pressure. However, a few studies have reported that signs of exaggerated [33] or decreased [34] sympathetic response at altitude are related to increased or decreased AMS, respectively [27, 35]. One hypothesis regarding the inhibition of AMS through altitude acclimatization involves downregulation of efferent renal sympathetic nerve activity so that the kidneys can sustain diuresis and prevent or limit the edema associated with high-altitude illness [36, 37]. Beidleman *et al*. [32] suggest that decreased or increased MABP in the present study may also be a marker of decreased or increased sympathetic activation, respectively.

The decrease in peripheral vascular resistance causes an increase in cardiac output, which is likely related to the decrease in DBP and the subsequent increase in pulse blood pressure (pulse BP). Nevertheless, the gradual decline in pulse BP from low altitude to high altitude may be due to an increase in vasomotor tone caused by the release of catecholamine. Although the altitude-induced increase in BP has been predominantly ascribed to sympathetic activation [2, 7, 38–42], there might also exist some other mechanisms, e.g., the activation of the renin-angiotensin system (RAS) and the release of vaso-active substances.

We also observed that high SBP and DBP were correlated with poor sleep quality. Numerous studies have established an association between insomnia and hypertension [43–47]. Insomnia is a common disorder characterized by subjective symptoms of problems in initiating or maintaining sleep, often associated with states of “hyperarousal”. Elevated BP occurring in association with insomnia may mirror the effects of sleep curtailment and/or sleep disruption on sympathetic activity [44]. Moreover, a report demonstrated that ESS was positively correlated with BP at all time points. In healthy older adults, Goldstein *et al*. predicted that, compared with individuals who showed few signs of daytime sleepiness, those who were sleepy during the day would have higher BP and would be more likely to develop hypertension after 5 years [48].

According to our findings, we also see that at high altitude, Post-exercise SBP and Pulse BP surpassed Pre-exercise SBP. On the one hand, this is due to the reinforcement of cardiac contractility and the increase of stroke volume, which result in high BP; on the other hand, the increase of skeletal muscle sympathetic activity could result in muscle contractibility during exercise at high-altitude 3,700 m, which causes some vasoconstrictive metabolites and Ang II to be produced. However, we did not find any changes in DBP. The reason may be that after exercise, heart rate increased, myocardial systolic time was shortened, and cardiac contraction was strengthened in order to ensure normal blood circulation. Most of the contractile force was used to pump the blood into the systemic circulation (SBP), while the absorption force of aortic dilatation (the DBP force) was relatively small. Therefore, SBP increased significantly after high-altitude exercise while DBP displayed no obvious changes.

### Limitations

There are several limitations to our study. One practical limitation was that our study was not an anterior-posterior self-control study. One other potential limitation is that BP responses in the current study were only observed in young male subjects, and the fact that BP remains elevated for longer than expected cannot be used to conclude how older people will respond over a similar time period. The BP response to high altitude of older travelers who might have baseline hypertension has recently been addressed by Luks [49]; however, some aspects of BP at high altitude are yet to be elucidated. Moreover, the lack of significance in the prevalence of AMS between men and women existed in a study when AMS severity was increased in men [50]. Although the existence of a sex difference in the context of this study is unclear, a sex difference does exist in AMS incidence [51]. Consequently, further studies incorporating female subjects need to be carried out to corroborate the current findings.

## Conclusions

Taken together, our study suggested that blood pressure did not manifest significant monotonic changes with time and increasing altitude. Moreover, there was a trend for higher blood pressure in the AMS group. And the degree of BP alteration appears to be related to the severity of the AMS symptoms (LLS), which suggest their utility in high-altitude clinical settings. Simultaneously, a higher BP response to hypoxia seems to identify subjects prone to develop AMS, and potentially, an exaggerated chemoreflex sympathetic vascular response is implicated in the genesis of AMS. Based on this study, higher BP at high altitude leads to lower sleep quality. BP should be considered as a parameter to be monitored in all adults who ascend to high elevations. Further studies are needed to take sex, age, and ethnicity into consideration.

## Abbreviations

AMS: acute mountain sickness
HA: high altitude
LA: low altitude
BP: blood pressure
SBP: systolic blood pressure
DBP: diastolic blood pressure
MABP: mean arterial blood pressure pulse
BP: pulse blood pressure
SO_2_: oxygen saturation
LLS: lake louise score
ESS: epworth sleepiness scale
AIS: athens insomnia scale
HACE: high-altitude cerebral edema
HAPE: high-altitude pulmonary edema
Ang II: angiotensin II
RAS: renin-angiotensin system.
